# Preparation and Hydration Properties of Steel Slag-Based Composite Cementitious Materials with High Strength

**DOI:** 10.3390/ma16072764

**Published:** 2023-03-30

**Authors:** Zhiming Xu, Ying Ma, Jiahao Wang, Xiaodong Shen

**Affiliations:** 1 College of Materials Science and Engineering, Nanjing Tech University, Nanjing 211816, China; 2 State Key Laboratory of Materials-Oriented Chemical Engineering, Nanjing Tech University, Nanjing 211816, China

**Keywords:** steel slag, solid wastes, compressive strength, hydration, microstructure

## Abstract

Steel slag (SS) has been largely discharged but little utilized, causing an environmental problem in China. In this paper, SS-based composite cementitious materials with high strength were prepared by the high volume of SS (≥40%), granulated blast-furnace slag (GBFS), fly ash (FA), flue gas desulfurization gypsum (FGDG) and cement to promote the effective utilization of SS. The hydration and hardening properties were studied through setting time, compressive strength, length change, isothermal calorimetry (IC), X-ray diffraction (XRD), mercury intrusion porosimetry (MIP), and scanning electron microscopy equipped with energy dispersive spectroscopy (SEM-EDS) tests. The results show that SS-based composite cementitious material exhibited a lower hydration heat release, an appropriate setting time, and volume stability. The SS cementitious material with 40% SS could obtain high strength of over 65 MPa at 28 days and 80 MPa at 90 days. The strength value of > 60 MPa is present in the binder, with 50% SS at 56 days. GBFS promotes hydration reactions and the formation of AFt and C-(A)-S-H gel, thus enhancing compressive strength. FA has a beneficial effect on later strength. The small and fine pore structures contribute to the development of strength. The main hydration products of SS composite cementitious materials are C-(A)-S-H gel, and ettringite (AFt), with less Ca(OH)_2_. The C-(A)-S-H gel with a lower Ca/Si ratio and a higher Al/Ca ratio in cementitious material, promotes mechanical properties.

## 1. Introduction

Steel slag (SS) is an industrial solid waste discharged during steelmaking. Its emissions were 160 million tons, and the utilization rate was less than 30% in China. A great part of SS is randomly discarded, resulting in land occupation and environmental pollution. The improvement of efficient utilization of SS is conducive to ecological environment protection and achieving sustainable development of the steel industry.

The mineral composition of SS contains C_3_S, γ-C_2_S, C_4_AF, C_2_F, RO phase (CaO–FeO–MnO–MgO solid solution), free-CaO, and free-MgO [1]. SS has been mainly used as aggregate substitution [2,3,4] and supplementary cementitious materials [5,6] in building materials. Some mechanical properties and durabilities of concrete with complete or partial substitution of SS aggregate were examined. The compressive strength of concrete would increase as the substitution ratio of SS aggregate replacing traditional aggregate increased [4,7,8,9]. There was a reduction in the compressive strength of concrete containing SS aggregates after freezing-thawing cycles due to high porosity [10]. It was proposed that the replacement ratio of SS aggregate at 15% and 30% are beneficial to the compressive strength [11]. 

SS has a low reactivity with respect to the high amount of γ-C_2_S resulting from the slow and natural cooling during SS production. Low reactivity calcium silicate in SS participates in cement hydration to generate hydrated calcium silicate. SS as supplementary cementitious materials could improve the workability, decrease hydration heat and decrease compressive strength [5]. The compressive strength decreases with the increase in the SS replacement ratio [12,13,14]. The 28-day compressive strength of mortar is about 37 MPa when the SS content reaches 30% [15]. 

It should be noted that the use of high-volume SS (≥40%) in steel slag-based cementitious material can efficiently promote the utilization of SS. Low compressive strength was reported in cementitious materials with high-volume SS. A low 28-day compressive strength of 30 MPa was exhibited in cement paste with 50% SS [16] and with the replacing ratio increase to 70%, the strength of cement paste was only 20.8 MPa [17]. He et al. [18] reported that 80% SS-20% cement paste has a 28-day compressive strength of about 35 MPa and decreases by 60 MPa compared with that of pure cement paste. 

Due to the low activity of SS, activation is necessary to promote the activity and hydration properties of SS-based composite binders. Alkali and calcium sulfur activation were primarily used in low-activity cementitious materials. Potassium and sodium compounds were commonly used as activators in alkali-activated cementitious materials. Under alkali conditions, the silicate and aluminate minerals dissolve and hydrate to produce C-S-H, C-A-S-H and N-A-S-H. A typical calcium sulfur-activated cementitious material (supersulfated cement) is composed of 70% to 90% slag, 0% to 5% Portland cement (clinker) or calcium hydroxide, and 10% to 20% sulfate activators (hemihydrate and gypsum dihydrate). The activity of SiO_2_ and Al_2_O_3_ is activated by calcium and sulfate and undergoes a hydration reaction to form C-(A)-S-H and AFt.

The steel slag-based composite cementitious material with a high volume of SS could be prepared based on the activity matching between the aluminate and silicate phases in SS, GBFS, FA with the calcium and sulfate phases in cement (clinker) and gypsum. It hydrates to generate C-S-H, Aft, or AFm, and potentially results in high cementitious properties. It was proposed that the addition of SS of less than 20% could slightly improve the compressive strength of SS-GBFS-FGDG mortar [19]. But a higher content of SS would have a negative impact on the compressive strength [19,20]. The hydration degree of the composite cementitious materials was improved by GBFS with higher activity, thereby improving the compressive strength. [16,21,22]. The compressive strength of the SS-FA-FGDG ternary system decreases with the substitution ratio of FA replacing SS. The strength of 60% SS-20%FA-20%FGDG paste is 30 MPa, and the strength decreases to 15 MPa while the replacing ratio of FA increases to 40% [23]. The previous studies indicate that composite cementitious materials can possess a higher strength with a lower volume of SS (≤20%), and for a high volume SS, low compressive strength occurred for SS-based composite cementitious material [24].

This study aims to prepare a high-strength SS-based composite cementitious material using high volume SS (≥40%) along with GBFS, FA, FGDG and less Portland cement. The hydration and hardening properties of SS-based composite cementitious materials were analyzed through hydration heat evolution, setting time, compressive strength, length change, XRD, MIP, and SEM-EDS tests. This study will further promote the effective utilization of steel slag, sustainable development of resources and environmental protection.

## 2. Experimental

### 2.1. Raw Materials

**Figure 1:**
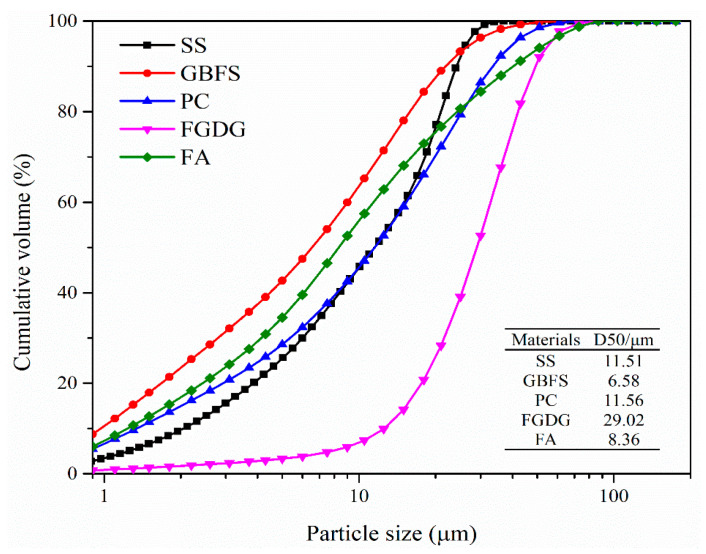
Particle size distributions of raw materials.

Fine powders of SS from Jigang Group Co., Ltd., Shandong, GBFS from Jintaicheng Environmental Resources Co., Ltd., Hebei, and low calcium FA were used in the experiment. Flue gas desulfurization gypsum was treated through a 200-mesh sieve in size of 75 μm. PC-II 52.5 Portland cement from Jiangnan Onoda was used. Particle size distributions of these fine materials were measured and shown in Figure 1. The medium diameter (D50) of SS, GBFS, PC, FGDG and FA were 11.51, 6.58, 11.56, 29.02, and 8.36 μm, respectively.

**Figure 2:**
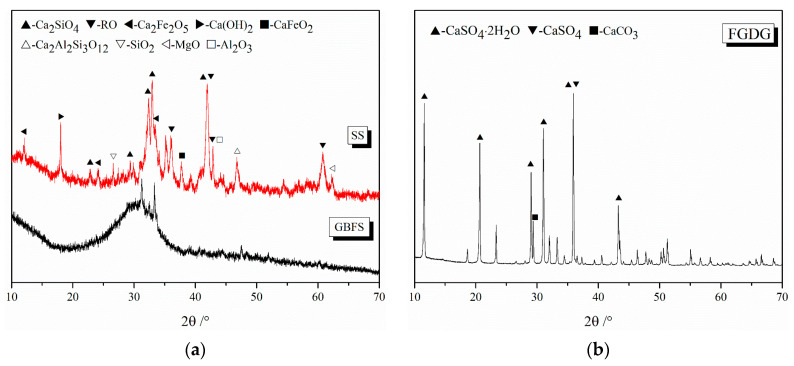

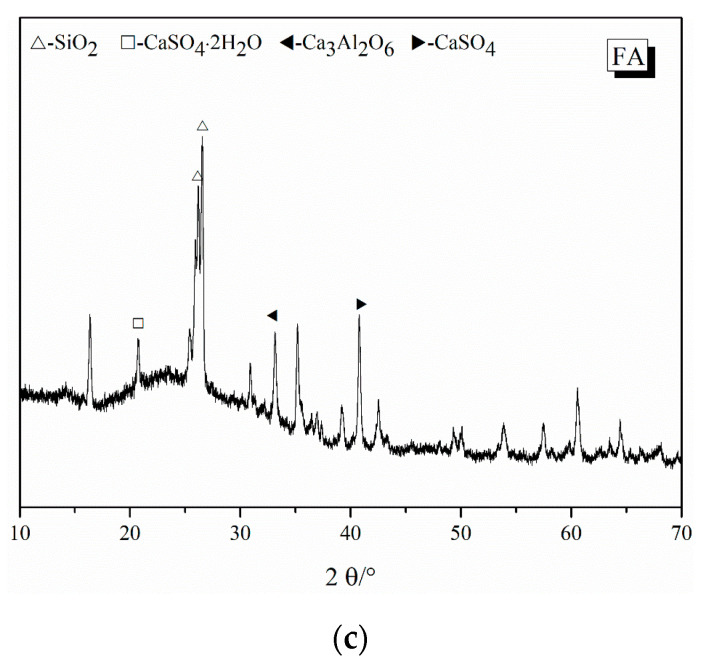
XRD patterns of (**a**) SS and GBFS, (**b**) FGDG and (**c**) FA.

The chemical and mineral compositions of raw materials were measured by X-ray fluorescence (XRF) and XRD as shown in Table 1 and Figure 2. The main chemical composition of SS is CaO, followed by Fe_2_O_3_ and SiO_2_, MgO, and MnO. Its mineral composition includes C_2_S, Ca_2_Fe_2_O_5_, RO and Ca(OH)_2_ phases. The alkalinity coefficient of the SS is 1.94 regarded as medium alkalinity according to Mason’s theory [25]. GBFS is composed by CaO and SiO_2_, followed by Al_2_O_3_ as well as the amorphous phase. Fly ash consists of SiO_2_, CaSO_4_, Ca_3_Al_2_O_6_, etc. The main mineral phases of FGDG were dihydrate (CaSO_4_·2H_2_O), anhydrite (CaSO_4_) along with calcium carbonate (CaCO_3_).

### 2.2. Preparation and Curing Procedures

**Figure 3:**
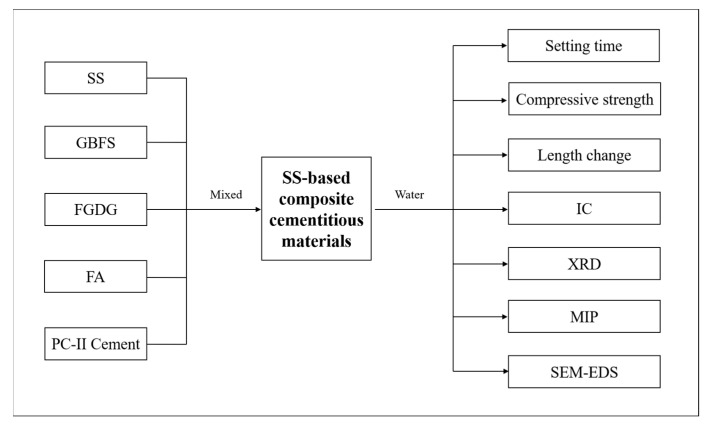
Experimental flowchart of this study.

The experimental flowchart of this study is shown in Figure 3. The mixture proportions of SS-based composite cementitious materials are shown in Table 2. The water-to-binder ratio of 0.3 was used. The fluidity of SS-based composite cementitious material is a range of 190–250 mm. The moulds in sizes of 20 mm × 20 mm × 20 mm and 20 mm × 20 mm × 80 mm were used for compressive strength and length change measurements, respectively. The raw materials were premixed, and the paste was mixed by a mixer for 4 min. The paste with the mould was vibrated by a vibrating machine. After casting, that was placed in a curing box (20 ± 2 °C and RH 95 ± 2%) for 24 h. Then, the cubes were demoulded and cured in water at 20 ± 1 °C until certain periods. At the ages of 3, 7, 14, 28, 56, and 90 days, parts of the samples were taken out and prepared for compressive strength and length change tests. Moreover, other parts of the samples were broken into pieces and soaked in anhydrous ethanol for 24 h [26] to stop the hydration. The sample pieces were dried in a vacuum oven at 40 °C for 24 h. The prepared sample pieces were used for MIP and SEM-EDS analysis. Parts of the sample pieces were ground and passed through a 200-mesh sieve for the XRD test.

### 2.3. Test Methods

The hydration heat evolution of SS-based composite cementitious materials was tested and recorded for 72 h using an isothermal calorimeter (TAM Air). The weight of 1.8 g of deionized water and 6 g of the binder were used. The slurry was mixed outside and immediately loaded into the isothermal calorimeter. The temperature was set and stable at 20 °C.

The fluidity of binders was tested according to GBT/2419-2016.

The initial and final setting times were conducted by using a Vicat apparatus according to the Chinese standard of GB/T 1346-2011.

The compressive strength was measured by a pressure testing instrument (AEC-201 type) with 2.4 KN/s according to JGJ/T 70-2009. The strength value was calculated by taking the average of at least four cubes. The length change was measured according to GB/T 29417-2012. The data on length change was collected by a digital comparator (precision of 0.001 mm). The average value of three cubes was used. The linear expansion ratio was obtained by calculating the length value at specific periods corresponding to the initial length value.

The hydration products of pastes were identified by XRD tests. XRD analysis was performed by a Rigaku-Smart-lab 3000A X-ray diffractometer with Cu Kα radiation at 35 mA and 40 kV. The scanning range is between 5° and 70° at 5° per minute with a step of 0.01°. 

The pore parameters of samples were measured by using a MIP (Auto Pore V 9600, Micromeritics). The pressure was 0.1 to 33,000 psi, and the pore size was in a range of 5.48 to 10,000 nm. 

The samples were coated with a gold layer for SEM-EDS measurement. The micromorphology of samples was observed by a ZEISS instrument (Ultra 55 FESEM) at the age of 90 days. Elements in the selected micro-regions were determined by the EDS at an accelerated voltage of 10 kV.

## 3. Results

### 3.1. Hydration Heat Evolution and Setting Time

**Figure 4:**
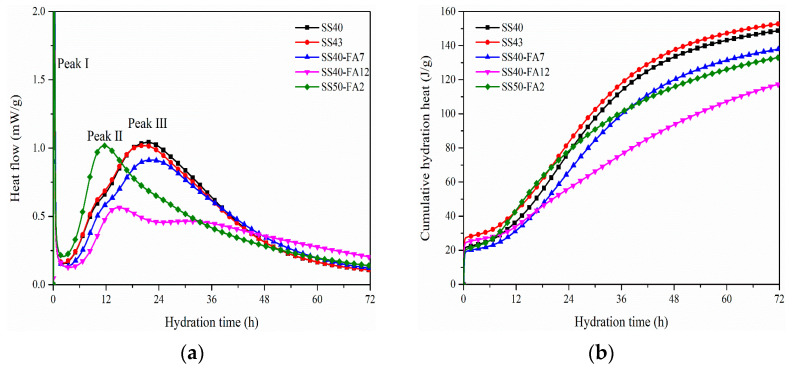
(**a**) Hydration heat flow and (**b**) cumulative hydration heat of SS-based composite cementitious materials.

The hydration heat flow and cumulative hydration heat of cementitious materials are shown in Figure 4. Three exothermic peaks were observed on heat flow curves for pastes except for the SS50-FA2 group. The first exothermic peak which corresponds to the dissolution of mineral ions and initial hydration of pastes is not accurate and fully displayed because of the limitation of the device and mixing way. The first exothermic peak was followed by an induction period with relatively small amounts of heat release and a short duration. The duration time shortens with the increase in SS. The heat flow then begins to enter the acceleration period, and the starting time is ordered by SS50-FA2 < SS43 and SS40 < SS40-FA7 < SS40-FA12.

A high exothermic peak II occurred in the SS50-FA2 paste. Other cementitious materials show a relatively lower exothermic peak II at around 10 to 14 h. The occurrence time of peak II is prolonged with the rise of FA content in SS40-FA7 and SS40-FA12 pastes compared with the SS40 group. The cement is replaced by SS with medium alkalinity, and the second exothermic peak appears around 10 h [27]. It is inferred that the second exothermic peak would be caused by the hydration of SS particles based on the high heat release at peak II for SS50-FA2 paste with a high content of SS.

The peak III appeared in SS40, SS43, and SS40-FA7 pastes after 17 h. SS40-FA12 paste presents a small peak III after 28 h resulting by a high addition of FA. The third exothermic peak is considered in relation to the main hydration of active mineral phases of binders as a result of the formation of C-S-H gel and Ca(OH)_2_ [28].

The cumulative hydration heat presents about 117.52–152.92 J/g for SS-based composite cementitious materials, which is lower than Portland cement systems. The cumulative hydration heat of SS43 paste at 1 and 3 days is slightly higher than that of SS40 paste. The cumulative hydration heat of SS40-FA7 and SS40-FA12 paste decreased with the increase of FA compared to SS40 at 1 and 3 days. It has been recognized that FA leads to a lower cumulative heat release than GBFS and Portland cement [29]. The fact that SS50-FA2 paste has a larger cumulative hydration heat than SS40-FA12 paste shows that SS releases more heat than FA. It is indicated that the activity of SS is higher than FA in cementitious materials at an early age.

**Figure 5:**
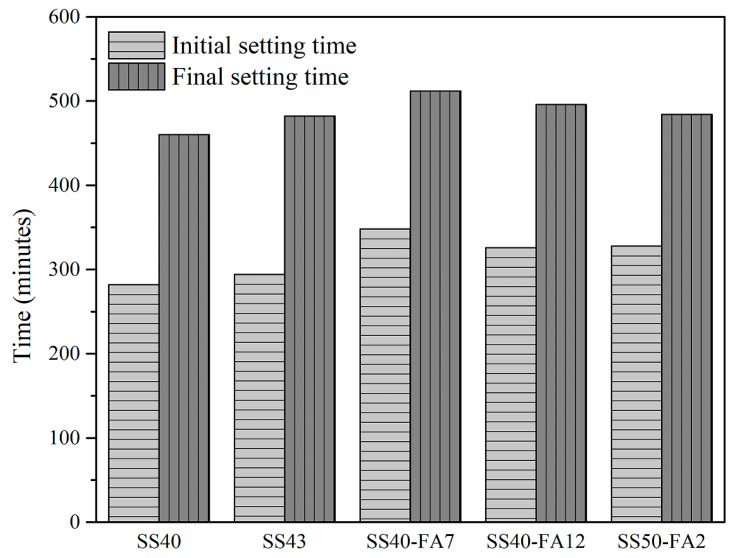
Initial and final setting times of SS-based composite cementitious materials.

The initial and final setting times are important indicators of early hydration. The initial and final setting times of pastes are shown in Figure 5. The initial and final setting times of pastes are at ranges of 282–348 and 460–512 min respectively, which meets the requirement of the standard of GB/T 1346–2011 [30]. Compared with SS40 paste, the initial and final setting times of SS40-FA7 and SS40-FA12 pastes were prolonged by the addition of FA. SS40-FA12 and SS50-FA2 pastes exhibited similar initial and final setting times. The proper setting time of steel slag paste facilitates the placement and handling.

### 3.2. Compressive Strength

**Figure 6:**
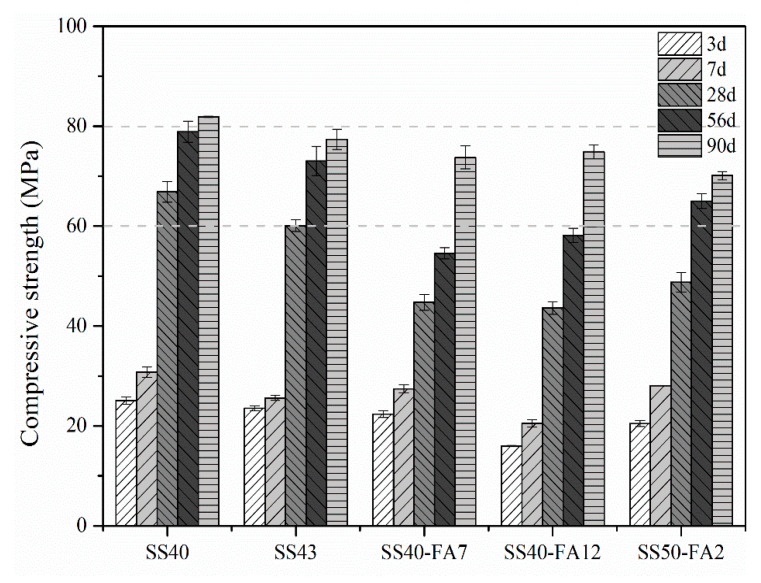
Compressive strength of SS-based composite cementitious materials at different ages.

Results of the compressive strength of cementitious materials at different ages are shown in Figure 6. It can be seen that the compressive strength increases as hydration time increases. The compressive strength of SS40 paste exceeds 60 MPa at 28 days and 80 MPa at 90 days, while the compressive strength of other pastes exceeds 70 MPa at 90 days. The SS40 specimen had higher compressive strength values. The continuous hydration of SS and GBFS increases the amount of C-(A)-S-H gel, which greatly improves the pore structure of pastes and results in the development of compressive strength.

Compared to the SS40 specimen, SS40-FA7 and SS40-FA12 pastes possessed lower compressive strength at all test ages because of the addition and the lower activity of FA in binders [31]. A high rise in compressive strength occurred for SS40-FA7 and SS40-FA12 pastes after 56 days, and the increase ratio is 35% and 29% from 56 to 90 days. It indicates that the FA plays an advantageous role in the development of compressive strength, due to the activation of the pozzolanic activity of the FA by the Ca(OH)_2_ formed in the paste at later ages. The C-S-H gel formed from the hydration of FA can fill the pores [32], resulting in a rapid rise in compressive strength at 90 days. The compressive strength of SS50-FA2 paste was higher than that of SS40-FA12 within 56 days, indicating that SS is superior to FA in the development of compressive strength. 

### 3.3. Hydration Products

**Figure 7:**
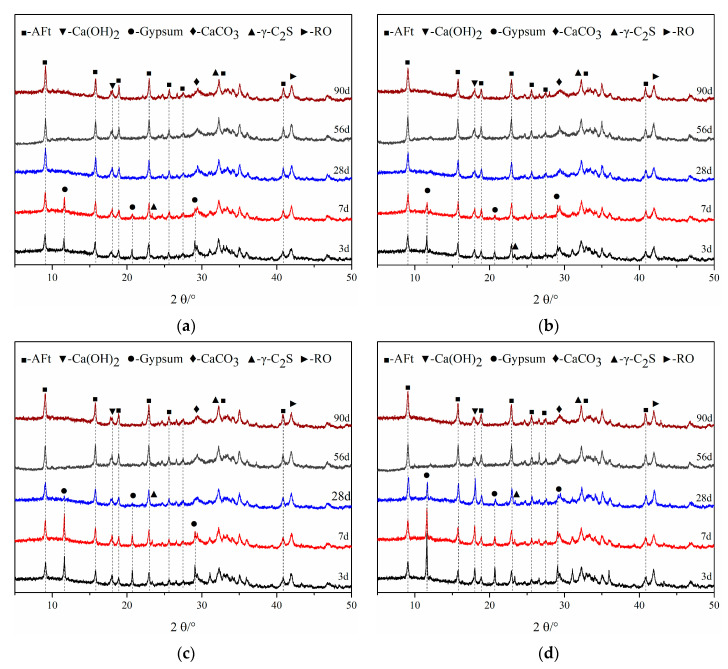

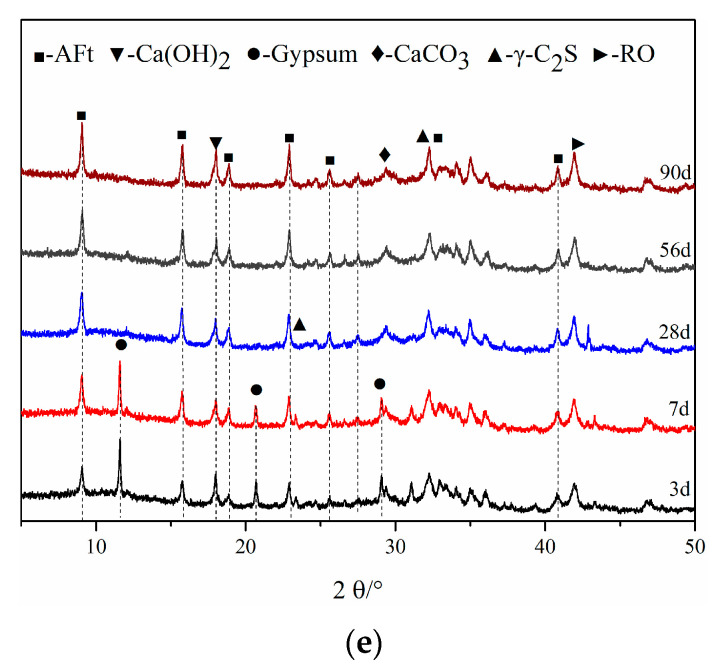
XRD patterns of (**a**) SS40, (**b**) SS43, (**c**) SS40-FA12, (**d**) SS40-FA12 and (**e**) SS50-FA2 at different ages.

The hydration products of all pastes at different ages (up to 90 days) determined by XRD are shown in Figure 7. It is observed that the phases, of AFt, Ca(OH)_2_, and various unhydrated phases including RO, γ-C_2_S, and gypsum, are produced in cementitious materials. A stronger intensity of AFt and a relatively weaker intensity of Ca(OH)_2_ are exhibited in SS cementitious materials, while amorphous C-S-H cannot be detected.

When the binders were mixed with water, cement minerals hydrated first and produced C-S-H gel and Ca(OH)_2_. The Si-O and Al-O bonds in GBFS, FA [33] and SS particles are decomposed in an alkaline environment to produce Ca^2+^, ${SiO}_{4}^{4-}$ and ${AlO}_{4}^{5-}$, respectively. These ions can participate in hydration reactions to form hydrated calcium silicate and hydrated calcium aluminate according to Equations (1) and (2). The latter can react with ${SO}_{4}^{2-}$ released from FGDG to produce AFt [20], as shown in Equation (3). The skeleton structure formed by AFt is conducive to the formation of early strength, and the continuous formation of C-S-H gel and AFt promotes the development of compressive strength.

(1) $${\mathrm{Ca}}^{2+}+x{\mathrm{SiO}}_{4}^{4-}+yH_{2}O\;\to \;\mathrm{CaO}\cdot x{\mathrm{SiO}}_{2}\cdot yH_{2}O$$

(2) $${5\mathrm{Ca}}^{2+}+{2\mathrm{AlO}}_{4}^{5-}+8H_{2}O\;\to \;3\mathrm{CaO}\cdot {\mathrm{Al}}_{2}O_{3}\cdot 6H_{2}O+2\mathrm{Ca}(\mathrm{OH}{)}_{2}$$

(3) $$3\mathrm{CaO}\cdot {\mathrm{Al}}_{2}O_{3}\cdot 6H_{2}O+3\mathrm{CaS}O_{4}+26H_{2}O\;\to \;3\mathrm{CaO}\cdot {\mathrm{Al}}_{2}O_{3}\cdot 3\mathrm{Ca}{\mathrm{SO}}_{4}\cdot 32H_{2}O$$

It can be seen that the diffraction peak intensity of AFt increases and the diffraction peak of gypsum gradually decreases with the development of hydration time, due to the consumption of gypsum during the formation of AFt. The disappearance of the gypsum diffraction peak can be observed in SS40 and SS43 pastes after 28 days. After 56 days, there were no obvious signs of a gypsum diffraction peak in other cementitious materials. The higher amount of GBFS in SS40 and SS43 pastes results in faster consumption of gypsum, which would be caused by a greater hydration reaction between the aluminate of GBFS and gypsum to produce AFt [34,35]. The gypsum diffraction peak of SS50-FA2 is lower than that of SS40-FA12 indicating that more steel slag leads to faster consumption of gypsum.

The diffraction peak of γ-C_2_S decreases with the hydration time. The presence of CaCO_3_ would be caused by carbonization, but most of it comes from FGDG. The diffraction peak of the RO phase remained and exhibited insignificant change up to 90 days.

### 3.4. Length Variation

**Figure 8:**
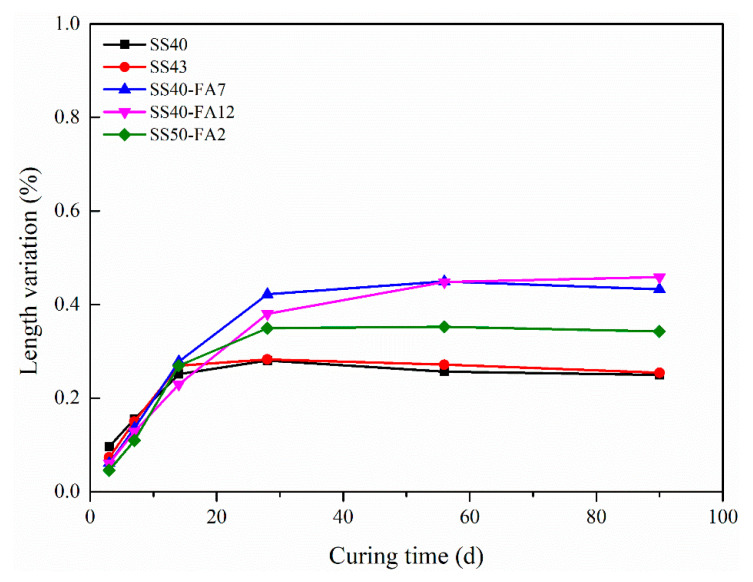
The linear expansion ratio of SS-based composite cementitious materials under water curing up to 90 days.

The linear expansion ratio of SS-based composite cementitious materials is presented in Figure 8. All pastes expended and the values of the expansion ratio are less than 0.46% and cured in water for up to 90 days. There is a rapid increase in the linear expansion ratio before 28 days and then a steady increase afterward. 

SS40 and SS43 pastes showed similar length changes, and the linear expansion ratios are less than 0.25%. It cannot be observed an increase in the expansion ratio up to 90 days when the amount of SS increases from 40% in SS40-FA12 to 50% in SS50-FA2. The potential volume instability has been proposed because the reaction between f-CaO and f-MgO in the SS and water produce Ca(OH)_2_ and Mg(OH)_2_ as a result of expansion [36]. The volume stability of SS cementitious materials exhibited a relatively weak variation with the rise of SS content up to 50% in this paper.

It can be seen that cementitious materials incorporating FA occurred a larger length change at 90 days. SS40-FA12 paste exhibited a higher expansion ratio than SS40-FA7 and SS50-FA2 specimens. The linear expansion ratio rises with the increase in FA content. The expansion development is dominated by AFt formation in the expansion performance of cement pastes with FA at early ages [37]. 

### 3.5. Pore Parameters

**Figure 9:**
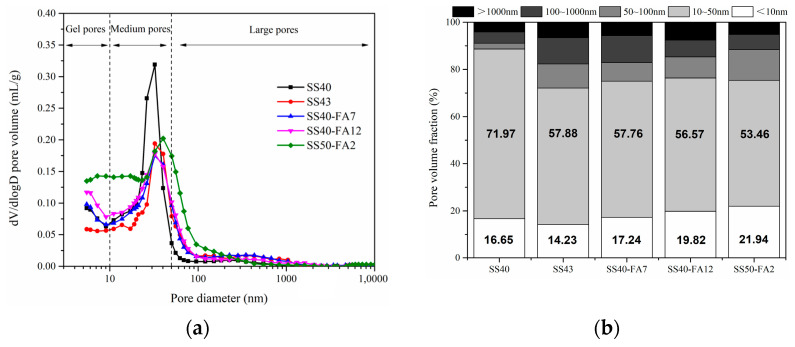
(**a**) Pore size distribution curves and (**b**) pore volume fractions of SS-based composite cementitious materials at 90 days.

The pore size distribution curves and pore volume fractions of cementitious materials are shown in Figure 9. Pore sizes smaller than 5.48 nm were not detected due to the limited method of testing. To gain more insights into the pore structure, the measured pore size distribution was divided into three size ranges: gel pores (<10 nm), medium pores (10–50 nm) and large pores (>50 nm) [38]. Table 3 summarizes the pore parameters including average and median pore diameter as well as the total porosity of specimens at 90 days. In general, the smaller the average pore size, the denser the pore structure, and the higher the strength [39]. Previous studies have shown that compressive strength is inversely proportional to the size of the porosity [40,41].

The gel and medium pores possess roughly 75%–89% of the volume fractions, meaning most occurrence pores in specimens (Figure 9). SS40 paste possesses a high-volume fraction of up to about 72% in the medium pore range, and the proportion of <50 nm pores accounts for 89%. The SS40 specimen exhibited a lower average pore diameter, and its porosity is 23.61%. It is predicted that its small and fine pores contribute to its high compressive strength (Figure 6). A large proportion of large pores and small porosity occurred in the SS43 paste. SS40-FA7 and SS40-FA12 specimens present similar pore parameters. A high fraction of gel pores but a low proportion of medium pores were observed in the SS50-FA2 paste. SS50-FA2 specimen has the smallest average pore size, but the highest porosity than other pastes. The lowest compressive strength would be mainly attributed to its high porosity.

### 3.6. Micromorphology

**Figure 10:**
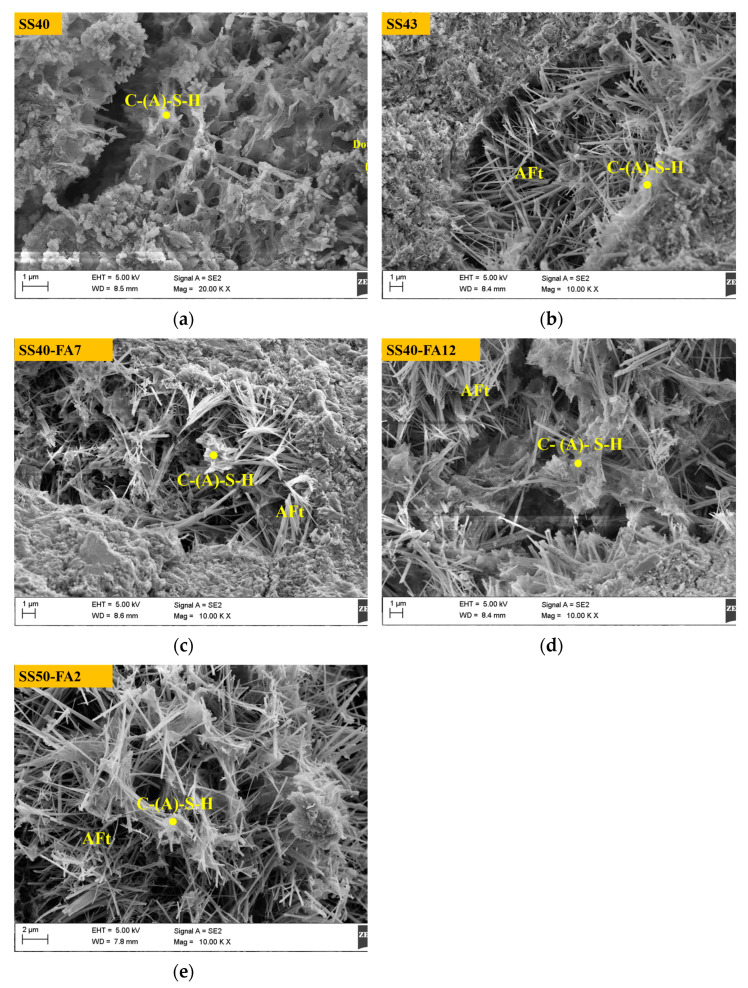
SEM images of SS-based composite cementitious materials at 90 days.

SEM micrographs of pastes at 90 days are shown in Figure 10. The presence of AFt needles with a width of around 200 nm and C-(A)-S-H gels was found in all pastes. The dot-like substances, which may be the initial C-S-H gel [28] were observed in the SS40 paste. The structure of AFt interlaced with C-(A)-S-H gels occurred in other samples. It can be observed the spots of net structure with regard to the interconnection between AFt and C-(A)-S-H in SS40-FA12 and SS50-FA2 samples. 

**Figure 11:**
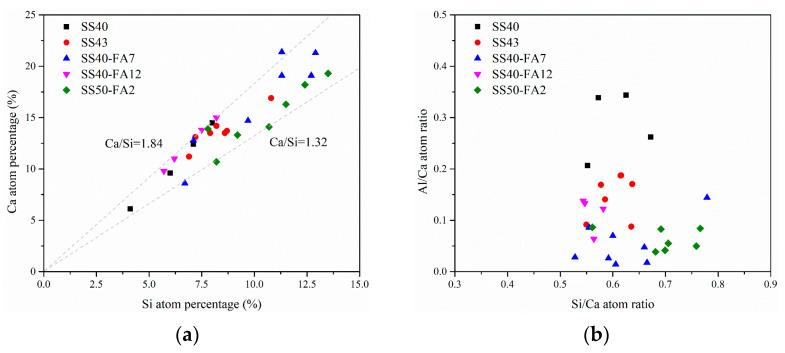
(**a**) Ca and Si atoms percentage, (**b**) Si/Ca and Al/Ca atomic ratios of C-(A)-S-H.

The percentages of Ca and Si atoms as well as Ca/Si and Al/Ca atomic ratios in C-(A)-S-H of pastes were explored by EDS analysis at 90 days, as shown in Figure 11. The Ca/Si ratio of C-(A)-S-H gel in cementitious materials is in the range of 1.32–1.84. Relevant works of literature [42,43,44] suggest that the Ca/Si of C-S-H for cement-based materials ranges from 1.50 to 2.58. SS cementitious materials exhibit a lower Ca/Si ratio of C-(A)-S-H compared to cement-based materials. The average silicate chain length of C-(A)-S-H increases with the decrease in the Ca/Si ratio [45]. Longer silicate chains contribute to the development of mechanical properties [43]. 

The Al/Ca ratio of pastes ranges from 0.02 to 0.34, and the average Al/Ca is 0.12 (Figure 11b). Al enters C-S-H mainly through silicic acid chain bridging sites [46], forming dense C-A-S-H gels [47]. The Al substitutes Si in C-S-H and high Al/Ca provide excellent mechanical properties [48].

## 4. Conclusions

In this paper, the high-strength SS-based composite cementitious materials were prepared by high volume SS (≥40%) along with GBFS, FA, FGDG and less Portland cement. 

SS-based composite cementitious materials exhibited a low hydration heat release. The second exothermic peak before the main peak on the heat flow curves is caused by the hydration of SS particles. The increase in SS or FA prolongs the initial and final setting times. The setting times of SS-based composite binders satisfy the requirement. 

The SS-based composite cementitious material (40% SS) could obtain a high compressive strength of over 65 MPa at 28 days and 80 MPa at 90 days. The strength value of >60 MPa is present in the binder with 50% SS at 56 days. GBFS with a higher activity promotes the hydration reaction and the formation of AFt and C-(A)-S-H gel, thus facilitating the compressive strength of the binder. The FA performs an advantageous effect on the strength at later periods. Meanwhile, the SS-based composite binders exhibited good volume stability.

The main hydration products of SS-based composite binders are AFt and C-(A)-S-H gel, with less Ca(OH)_2_. The network structure of the interconnection between the needles AFt and C-(A)-S-H gel formed, which is conducive to the dense structure. A lower Ca/Si ratio and a higher Al/Ca ratio of C-(A)-S-H gel formed, which promotes mechanical properties. 

Small and fine pores contribute to the high compressive strength of paste with 40% SS, and a higher SS volume (50%) would lead to an increase in porosity, resulting in a reduction in the strength of the paste (SS50-FA2). 

The results could provide a reference for the preparation and application of high-strength steel slag-based composite cementitious materials, which facilitate the efficient utilization of steel slag.

## Figures and Tables

**Table 1:** Chemical composition of raw materials (wt. %).

Materials	CaO	SiO_2_	Al_2_O_3_	SO_3_	MgO	Fe_2_O_3_	Na_2_O	K_2_O	MnO	P_2_O_5_	LOI
SS	36.46	16.64	5.71	0.24	7.42	21.03	0.29	0.09	6.06	2.14	1.06
GBFS	40.38	30.42	16.74	1.34	7.56	1.24	-	0.43	0.23	0.14	-
PC	62.13	21.75	5.21	1.97	2.09	2.91	-	0.63	0.05	0.16	2.40
FGDG	32.06	1.65	0.80	42.46	0.70	0.20	-	0.08	0.02	0.02	21.59
FA	7.95	46.41	31.18	1.91	1.30	4.97	0.60	0.86	0.08	0.60	2.40

**Table 2:** Mixture proportions (wt. %) of SS-based composite cementitious materials.

Binders	SS	FGDG	PC	FA	GBFS
SS40	40	13	15	0	32
SS43	43	13	15	0	29
SS40-FA7	40	13	15	7	25
SS40-FA12	40	13	15	12	20
SS50-FA2	50	13	15	2	20

**Table 3:** Pore parameters of SS-based composite cementitious materials at 90 days.

Groups	Average Pore Diameter (nm)	Median Pore Diameter (Volume) (nm)	Porosity (%)
SS40	19.07	27.77	23.61
SS43	23.10	34.35	20.91
SS40-FA7	20.60	31.00	22.84
SS40-FA12	19.19	28.91	23.37
SS50-FA2	18.35	27.29	30.28

## Data Availability

Data will be made available on request.
